# Hierarchical classification strategy for Phenotype extraction from epidermal growth factor receptor endocytosis screening

**DOI:** 10.1186/s12859-016-1053-2

**Published:** 2016-05-03

**Authors:** Lu Cao, Marjo de Graauw, Kuan Yan, Leah Winkel, Fons J. Verbeek

**Affiliations:** Imaging and Bio-informatics group, LIACS, Leiden University, Niels Bohrweg 1, Leiden, 2333 CA The Netherlands; Division of Toxicology, LACDR, Leiden University, Einsteinweg 55, Leiden, 2333 CC The Netherlands; Biomechanics Laboratory, Erasmus MC, Wytemaweg 80, Rotterdam, 3015 CN The Netherlands; The Department of Anatomy and Embryology, LUMC, Einthovenweg 20, Leiden, 2333 ZC The Netherlands

**Keywords:** Phenotype measurement, Image analysis, Wavelet-based texture measurement, Hierarchical classification, EGFR endocytosis, High throughput

## Abstract

**Background:**

Endocytosis is regarded as a mechanism of attenuating the epidermal growth factor receptor (EGFR) signaling and of receptor degradation. There is increasing evidence becoming available showing that breast cancer progression is associated with a defect in EGFR endocytosis. In order to find related Ribonucleic acid (RNA) regulators in this process, high-throughput imaging with fluorescent markers is used to visualize the complex EGFR endocytosis process. Subsequently a dedicated automatic image and data analysis system is developed and applied to extract the phenotype measurement and distinguish different developmental episodes from a huge amount of images acquired through high-throughput imaging. For the image analysis, a phenotype measurement quantifies the important image information into distinct features or measurements. Therefore, the manner in which prominent measurements are chosen to represent the dynamics of the EGFR process becomes a crucial step for the identification of the phenotype. In the subsequent data analysis, classification is used to categorize each observation by making use of all prominent measurements obtained from image analysis. Therefore, a better construction for a classification strategy will support to raise the performance level in our image and data analysis system.

**Results:**

In this paper, we illustrate an integrated analysis method for EGFR signalling through image analysis of microscopy images. Sophisticated wavelet-based texture measurements are used to obtain a good description of the characteristic stages in the EGFR signalling. A hierarchical classification strategy is designed to improve the recognition of phenotypic episodes of EGFR during endocytosis. Different strategies for normalization, feature selection and classification are evaluated.

**Conclusions:**

The results of performance assessment clearly demonstrate that our hierarchical classification scheme combined with a selected set of features provides a notable improvement in the temporal analysis of EGFR endocytosis. Moreover, it is shown that the addition of the wavelet-based texture features contributes to this improvement. Our workflow can be applied to drug discovery to analyze defected EGFR endocytosis processes.

## Background

The epidermal growth factor receptor (EGFR) is important for normal growth and function of breast tissue. Its signaling is regulated via endocytosis, a process that results in receptor degradation and thereby attenuation of the EGFR signaling. In cancer cells, however, the endocytosis pathway is often defected, resulting in an uncontrolled EGFR signaling. This uncontrolled EGFR signaling triggers breast cancer cells to escape from a primary tumor and spread to the lung, resulting in a poor prognosis for the disease progression. Moreover, it may result in complications like resistance to anti-cancer therapy.

**Fig. 1:**
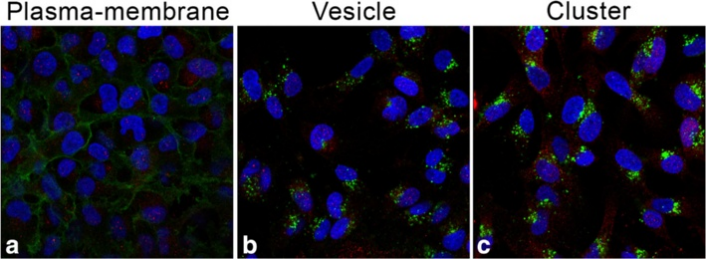
Sample images of the 3 phenotypic groups. *Red channel* is a P-ERK expression staining (Cy3); *green channel* is an EGFR expression staining (Alexa-488); *blue channel* is a nuclear staining (Hoechst #33258)

From the literature [1] a generic model of epidermal growth factor induced (EGF-induced) EGFR endocytosis can be divided into four characteristic episodes. (1) Under normal conditions, EGFR is localized at the plasma-membrane site for internalization; in our study this is defined as the “membrane” episode (membrane-episode). (2) Upon binding of EGF to the receptor, EGFR is taken up into small vesicular structures and starts sorting in early endosomes; in our study this is defined as the “vesicle” episode (vesicle-episode). (3) Over time, EGFR containing vesicles are transported to late endosomes localized near the nuclear region and these form into a larger complex multi-vesicular body; in our study this defined as the “cluster” episode (cluster-episode). (4) In final episode, EGFR is degraded in the lysosomes. In addition to this route, EGFR can also be partly transported back to the plasma-membrane sites. This dynamic model is used as the major guideline for the analysis of the EGFR-regulation-related genetic pathway using microscopy images as a readout. In this manner the analysis is linked to the analysis of the characteristic episodes in EGFR endocytosis. In this paper, we will focus our analysis on the aforementioned dynamic model, however, for the analysis we will only use the first three characteristic episodes, as shown in Fig. 1; the final episode of the EGFR signaling can not be visualized through markers in microscopy.

Over the past years, RNA interference in combination with fluorescence microscopy-based imaging has become a powerful tool for the visualization and high-throughput analysis of the complex EGFR endocytosis processes [2–4]. With these techniques, it becomes feasible to distinguish characteristic episodes and identify potential EGFR endocytosis regulators. It is, however, impossible to perform analysis through manual processing of the large volume of data that result from such high-throughput experiments. An automated method for the analysis of EGFR endocytosis is required [5]. To that end, through microscopy images are acquired and from these images features are extracted; combinations of these features should be characteristic for the episodes from the dynamic model that we use.

In the solution presented here, a single-step multi-class classification solution is demonstrated to properly capture the EGFR dynamics which transform along the three characteristic episodes and classify different EGFR episodes. From earlier applications we have identified some weaknesses for which we propose better solutions. First, the same subset of features is used to classify the three episodes. Second, a flat classification ignores the existence of potential hierarchical relationships that may exist in the data set. Fluorescence is used as a readout from the the images. Thus, the average intensity is used as a measurement for the phenotype. Variation of the fluorescent intensity in the images in the datasets is always present, and this variation complicates the classification. Consequently, a more advanced classification strategy is required.

Moreover, from observations in our previous results, we could determine that the vesicle-episode and cluster-episode have more morphological similarity with each other than with membrane-episode. In order to support this finding, we designed a new hierarchical classification strategy [6]. Hierarchical classification strategies are an efficient way to deal with complex classification problems. The problem is divided in an hierarchical manner so that classes that are more similar to each other are grouped together into a super class, thereby providing a simplification of original problem [7]. Now, in the hierarchical tree, each parent node has an individual classification scheme choosing related features and the best classifier to distinguish the child nodes. A hierarchical classification strategy specifically separates classifier training into two levels. In our case, for the first level we train a local classifier to distinguish the membrane-episode from the endosome, i.e. the super class containing vesicle-episode and cluster-episode. Subsequently, we train a second level local classifier to separate vesicle-episode from cluster-episode. With this strategy, we can make better use of the prominent features in the subsets and thereby, noticeably, improve the performance of the classifier. In addition, instead of only using the average intensity, we introduce a set of texture measurements. These texture features including texture features obtained from the wavelet transform in order to be able to describe the intensity characteristics in a more sophisticated way.

## Methods

### Cell material and preparation

The study of cell systems at the cellular level at large scale is referred to as cytomics. In high-throughput screening images are used as a readout for phenotyping. A common workflow for image data preparation in cytomics includes three essential steps: (1) cell culturing, (2) labeling, preparation for imaging and (3) image acquisition.

In this paper we use an EGFR-regulation related siRNA screening to illustrate this workflow. For the experiment, breast cells from a human breast carcinoma cell line (HBL100) were cultured in 96 well culture plates and transfected using Dharmafect smartpool Small interfering RNAs (siRNAs). Subsequently, the transfected cell population was exposed to epidermal growth factor (EGF) for a specific duration of time. Cells were fixed at different time points and visualized as a confocal slice with a confocal laser microscope (Nikon TE2000).

### Image acquisition and processing

Automated image acquisition was realized with a motion controlled microscope stage equipped with an auto-(re)focusing module. For each well, images were captured from ten randomly selected locations. For each image three channels were captured: (1) the red channel containing staining of Phospho-ERK(P-ERK) expression, i.e. Cy3 label, (2) the green channel containing EGFR expression staining, i.e. Alexa-488 label, and (3) the blue channel containing a nuclear staining, i.e. Hoechst #33258.

The subsequent image processing consists of two major steps: pre-processing and feature extraction including image segmentation. In the pre-processing step, the goal is optimizing the image for segmentation and subsequent analysis. For the type of images in our study two issues complicate the further processing, i.e. uneven background and noise as a result of the photo-multiplier in the confocal imaging. The rolling ball background subtraction method is used to remove large spatial variations of the background intensities [8]. Subsequently, a small Gaussian kernel is used to remove the noise from the image. The process of image segmentation refers to the process of partitioning an image into (multiple) regions of related content with the goal to simplify and/or change the representation of an image into components that can be measured. For fluorescence cell imaging, we utilized a customized segmentation algorithm known as the watershed masked clustering (WMC [9]). The WMC algorithm is an innovative segmentation algorithm that is particularly suitable for images in which the individual objects exhibit a variation in fluorescence. The WMC algorithm has been successfully applied in different types of cytomics studies like dynamic cell migration analysis [10, 11] and protein signaling modeling [12]. The WMC produces binary masks of the objects, in our case vesicles, as output. These binary masks are used to derive a number of phenotypic measurements for further data analysis. These measurements are applied for each of the channels in the image.

### Phenotype measurement

A phenotype is considered as the composite of the observable characteristics of an organism or traits: such as its morphology or developmental state. It is important for the detection of genetic variants in complex traits. Therefore, researchers should be aware of the theoretical importance of unbiased, reliable and replicable measurements [13]. In our previous work, we have already introduced an amount of basic measurements and localization phenotype measures [14]. In order to attempt finding more prominent phenotype measurements to characterize the three EGFR phenotypes, two aspects were considered. On the one hand, the phenotype measurements should be representative and relevant. On the other hand, these measurements must be robust to small variations in fluorescent intensity, meaning that the measurements are scale-free and self-normalized.

Based on the empirical observations in a ground truth data set, several potential texture patterns in object intensities were identified to characterize EGFR episodes. For instance, the vesicle-episode (2nd episode) has a higher intensity in the central region and relatively lower intensity around the vesicle-boundary. This is in contrast with the cluster-episode (3rd episode)which express a more evenly distributed intensity throughout the region of interest. In addition, these three EGFR episodes could also present distinctively in different texture features. Therefore, we introduced several texture measurements to describe the different phenotypical characteristics.

#### Texture measurement

For texture measurements the First Order Statistics are the most frequently used approach. These are derived from statistical properties of the intensity histogram of the image [15]. We used the standard First Order Statistics for each individual object as obtained from the segmentation; i.e. standard deviation of intensity, smoothness, skewness, uniformity and entropy. Definitions and formulations of these texture measurements are presented in Table 1.

**Table 1:** Description of phenotype texture measurements

Feature name	Expression	Description
std	$f_{1}=\sqrt {\sum \limits _{i}(i-mean)^{2}H\,(i)}$	The standard deviation of
		intensity from all the pixels
		in a region.
Smoothness	$f_{2}=1-\frac {1}{(1+{f_{1}^{2}})}$	The relative smoothness of the
		intensity in a region. It is 0 for a
		region of constant intensity and
		1 for a region with large excursion
		in the values of its intensity levels.
Skewness	$f_{3}=\sum \limits _{i}(i-mean)^{3}H\,(i)$	The order moment about the
		mean. The departure from
		symmetry about the mean
		intensity. It is 0 for symmetric
		histograms, positive for
		histograms skewed to the right
		and negative for histograms
		skewed to the left.
Uniformity	$f_{4}=\sum \limits _{i}H^{2}\,(i)$	The sum of squared elements in
		Histogram. It reaches maximum
		when all intensity levels are equal
		and decreases from there.
Entropy	$f_{5}=-\sum \limits _{i}H(i)\log _{2}{H\,(i)}$	The statistical measure of
		randomness.

i represents the intensity value

H(i) is the histogram of intensity

mean symbolizes the average intensity

#### Wavelet texture measurement

In addition to the standard texture features, recently, texture analysis based on the discrete wavelet transform (DWT) has been described [4, 16]. The wavelet texture features have shown to be an efficient descriptor for phenotyping [17], therefore we want to assess their feasibility in our framework. The DWT provides a set of texture representations consisting of coefficients in different directions. We calculated our wavelet-based texture measurements by multiplying each direction detail with the binary mask as obtained from the segmentation and henceforth calculating the mean, standard deviation and entropy of intensity for each labeled object in each direction details (see in Table 2). In this study, we included a biorthogonal wavelet [18] because it has the property of exact reconstruction and it is an outstanding wavelet representation for image decomposition. After decomposition, it generates the coefficient matrices of the level-one approximation and horizontal, vertical and diagonal details. Subsequently, we reconstructed the level-one details respectively from the corresponding coefficients. In this way, the texture details from three different directions are derived on the same scale as the original image.

**Table 2:** Description of Wavelet texture measurements

Feature name	Description
H_mean	The average intensity of Horizontal detail from discrete
	wavelet transformation.
H_std	The intensity variation of Horizontal detail from discrete
	wavelet transformation.
H_Entropy	The statistical randomness of Horizontal detail from
	discrete wavelet transformation.
V_mean	The average intensity of Vertical detail from discrete
	wavelet transformation.
V_std	The intensity variation of Vertical detail from discrete
	wavelet transformation.
V_Entropy	The statistical randomness of Vertical detail from discrete
	wavelet transformation.
D_mean	The average intensity of Diagonal detail from discrete
	wavelet transformation.
D_std	The intensity variation of Diagonal detail from discrete
	wavelet transformation.
D_entropy	The statistical randomness of Diagonal detail from
	discrete wavelet transformation.

### Production of ground truth data

For a supervised classification the collection of objective and sufficient ground truth data is important. We use the ground truth data as our training set during the classifier training.

**Fig. 2:**
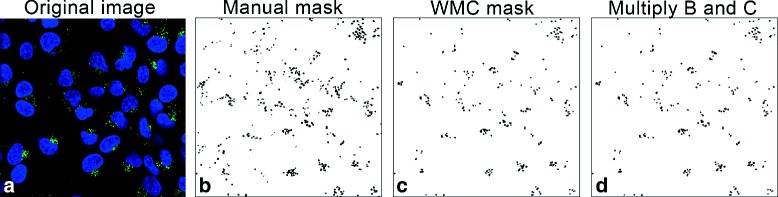
Ground truth data production

The ground truth data were obtained by outlining the three characteristic episode groups, i.e. membrane-episode, vesicle-episode and cluster-episode. These were separately outlined by domain specialist researchers using our annotation software (TDR); the outlining process is done with a digitizer tablet (WACOM, Cintiq LCD-tablet) [19] which is integrated in the software. From each outline a binary mask was created for each phenotypic stage. Figure 2b illustrates the vesicle-episode mask derived from a manually selected vesicle-episode outline. This mask was multiplied with the mask obtained from the WMC algorithm so as to extract the intersection (cf. Fig. 2d). Finally, the phenotype measurements were computed from these masks. The ground truth datasets for the membrane and cluster episodes were prepared in a similar manner. The training dataset included the three characteristic episode groups with a total of 2162 segmented objects from the images, i.e. 103 objects from cluster-episode, 374 objects from membrane-episode and 1685 objects from vesicle-episode; per object 25 features were used. The ground truth set is composed such that for each of the episodes sufficient objects, i.e. vesicles, are outlined to guarantee capturing the variation of the features in each of the episodes.

### Hierarchical classification strategy workflow

**Fig. 3:**
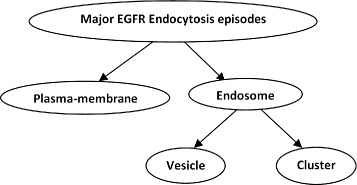
Hierarchical tree of EGFR endocytosis process

**Fig. 4:**
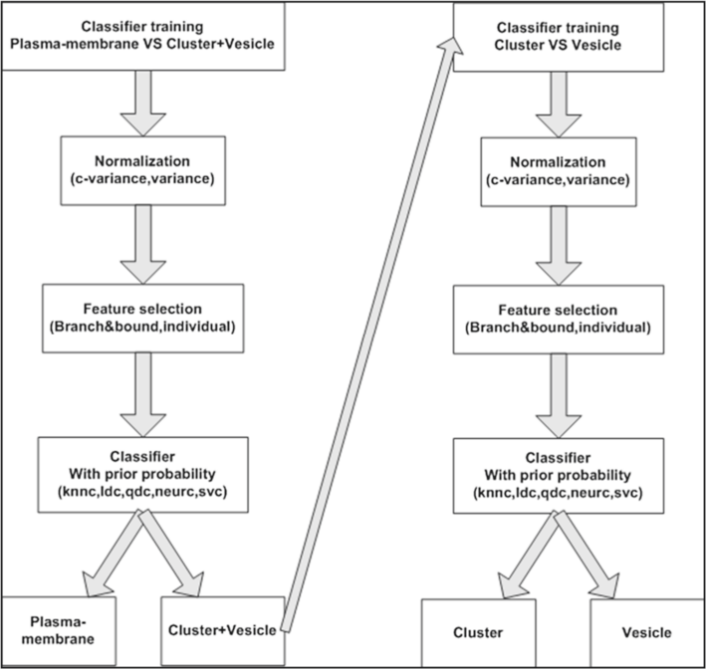
Hierarchical classification workflow

In the three characteristic episodes of the generic model of epidermal growth factor induced EGFR endocytosis that we use for our analysis we state that the vesicle-episode comprises single/early endosomes and the cluster-episode comprises clustered/late endosomes. Compared to the membrane-episode, these two episodes have more morphological similarity with each other. The vesicle-episode and cluster-episode episodes are located in the cytoplasm which have evenly distributed high intensity value and relatively circular shape. The membrane-episode is located around cell membrane which has an elongated shape with a low and unevenly distributed intensity value. Therefore, we have constructed the three characteristic episodes into a hierarchical tree as shown in Fig. 3. Subsequently, we use a local classifier per parent node approach to train a two-class classifier for each parent node in the class hierarchy. In doing so, the problem of making inconsistent predictions is avoided while, at the same time the natural constrains of class membership are taken into account [6, 20, 21]. In this manner, both the best classifier and the most prominent features are selected for each parent node classifier so as to classify the dynamic model with three episodes in a better fashion. The workflow of the hierarchical classification strategy is shown in Fig. 4. In our workflow, we normalized the dataset per feature, performed feature selection, applied the classifier and calculated the weighted classification error in order to evaluate the performance of the classification. We look for the best combination of classification process according to the error estimation and use this for EGFR episode classification.

#### Feature normalization

The features can have rather substantial differences in their dynamic range. Such is the case with the features that we use in this setup. Therefore, it is necessary to normalize our dataset. A feature normalization is required to approximately equalize ranges of the features and make them have roughly the same effect in the computation of similarity [22]. The main advantage of the normalization is to avoid dominance of attributes with larger numerical ranges over those with smaller numerical ranges. An additional advantage is that numerical complications during the computations are avoided; as kernel values depend on the inner products of feature vectors, large attribute values might introduce numerical complications [23].

We applied two types of normalization schemes to normalize the dataset. One standard normalization scheme was accomplished by shifting the mean of the dataset to the origin and scaling the total of variance for all features to 1, thereby, neglecting class priors. The other scheme was achieved by shifting the mean of the dataset to the origin and normalizing the average class variances (within-class). Class priors were taken into account. Moreover, the recently introduced concept of within-class covariance normalization for the support vector machine (SVM) classifier was employed [24]. For the evaluation of the methods considered in this paper, we are evaluating these two normalization schemes and we are interested to see whether, in our case, within-class covariance normalization outperforms the standard normalization. We will benefit from the fact that the normalization prevents differences in numerical scales.

#### Feature selection

After normalization, we applied a feature selection procedure. For feature selection a metric is required that considers a strong correlation among the variables. To that end the Mahalanobis distance [25, 26] was chosen. Subsequently, we selected two representative search algorithms: the branch and bound procedure [27] and best individual-N features. Branch and bound is a top-down procedure, beginning with the set of variables and constructing a tree by successively deleting variables. This feature selection method already showed a robust and good performance in our previous study [14]. The best individual-N features procedure is a computationally efficient method for choosing the best N features by assigning a discrimination power estimate to each of the features in the original set. This method could have a well-defined feature set when the features are uncorrelated. We will further use these two search algorithms in the feature selection part.

#### Prior probability setting

In probability theory and applications, the Bayes’ theorem shows the relation between posterior *P*(*A*|*B*), likelihood *P*(*B*|*A*) and prior *P*(*A*), expressed as:

(1) $$ P(A|B)=\frac{P(B|A)P(A)}{P(B)}  $$

A prior probability P(A) is the probability distribution of A before the specific condition is taken into account [28, 29]. It denotes attributing uncertainty rather than randomness to the quantity under investigation. A prior is often given by an expert and can be a purely subjective assessment or an estimation from objective observations. In order to obtain a prior knowledge for our application, we have chosen a group of images with 6 different time stages and manually counted the number of the three characteristic episodes. Subsequently, we calculated the ratio between membrane-episode and the super class (cluster-episode and vesicle-episode) as 0.0526 and the ratio between cluster-episode and vesicle-episode as 0.0556. These ratios are confirmed through observations by domain specialists. In our evaluations, we verified the performance of this prior probability with no prior probability and with equal prior probability. For the setting no prior, meaning an empty prior, it is assumed that the class prior probabilities correspond to the class frequencies in the dataset [30].

For our optimization scheme, the within-class co-variance normalization was selected. For the evaluations of the classifiers, the weighted error of different classifiers was calculated after a branch and bound feature selection. The weighted error estimation is defined as:

(2) $$ E=\sum_{k=1}^{n}{w_{k}*(e_{k}/n_{k})}  $$

where *e*_*k*_ represents the number of mis-classified objects of class k; *n*_*k*_ is the total size of class k; the weight *w*_*k*_ here is the prior probability of class k; *n* is the total number of classes in the dataset. From the results, as shown in Table 3, it can be perceived that including a prior probability results in an increase of the classifier performance.

**Table 3:** Prior probability comparison

C-variance (branch & bound)										
Equal prior	knnc		ldc		qdc		neurc		svc	
	mean	std	mean	std	mean	std	mean	std	mean	std
1st step	0.0333	0	0.095	0.0224	0.0317	0.0075	0.0633	0.0149	0.0333	0
2nd step	0.0575	0.0335	0.0575	0.0335	0.15	0	0.055	0.0224	0.1025	0.0112
No prior	knnc		ldc		qdc		neurc		svc	
	mean	std	mean	std	mean	std	mean	std	mean	std
1st step	0.0181	0.0014	0.0365	0.0057	0.0221	0.0037	0.01	0.0031	0.0142	0.001
2nd step	0.0292	0	0.0357	0.0088	0.043	0.0121	0.0348	0.0065	0.0402	0.0069
With prior	knnc		ldc		qdc		neurc		svc	
	mean	std	mean	std	mean	std	mean	std	mean	std
1st step	0.0053	0.0011	0.0061	0.0038	0.0059	0.0028	0.0061	0.0029	0.0061	0.0038
2nd step	0.0214	0.0031	0.0321	0.0011	0.056	0.0019	0.0231	0.0048	0.0214	0.002

#### Classifier

The classifiers in our evaluation have been selected to be able to cover both linear and non-linear categories; i.e. the linear classifier (LDC), the quadratic classifier (QDC), the K-nearest neighbor classifier (KNNC), the support vector machine classifier (SVC) and the neural network classifier (NEURC). Compared to linear classifiers, nonlinear classifiers are preferred for data that exhibit strong non-Gaussian distributions [31]. The linear classifier makes a classification decision based on the value of a linear combination of the characteristics [32]. The quadratic classifier is generalized form of the linear classifier in that it separates classes on the basis of a quadratic hyperplane. The K-nearest neighbor classifier distinguishes an object by majority voting of its neighbors, with the object being assigned to the class that is most common among its neighbors. The Support Vector Machine (SVM) is primarily a classifier method that performs classification tasks by constructing hyperplanes in a multidimensional space that separates cases of different class labels [33]. Key to the SVM is the use of kernels, the absence of local minima and the capacity control obtained by optimizing the margins [34].

In a neural network, units (neurons) are arranged in layers and these layers convert an input vector into some output. Each unit takes an input, applies a (often nonlinear) function to it and then passes the output on to the next layer [35]. There are many artificial neural network (ANN) models; i.e., Feed-Forward Networks, Radial Basis Function Networks, Recurrent Networks, etc. The advantage of neural networks is two-fold. First, neural networks are data driven self-adaptive methods. The flexibility is created by the combination of different nodes with related kernels. Second, they are universal functional approximators in which neural networks can approximate any function with arbitrary accuracy. The disadvantage of neural networks is that they are notoriously slow and it is rather difficult to determine the optimal number of kernel types, layers and nodes [36]. In this study we used the biologically inspired feed-forward neural network with a single hidden layer. The feed-forward neural network is defined as a unit feeding its output to all the units in the next layer, but there is no feedback to the previous layer. It is the simplest form of artificial neural network and it can yet limit the complexity of network calculations.

## Results and discussion

The results from our study are threefold. We first present the analysis of the different computational approaches that we have evaluated to come to the best possible classifiers for the Phenotype classification in our study. Moreover, we demonstrate that the hierarchical strategy results in the best overall classification. Subsequently, we explain the results of the application of the hierarchical classification scheme for an EGFR regulator knock-down study. Next to this study, the analysis was used in a study on the effect of EGF in a limited time-window. The results demonstrate the potential of the approach taken.

***Analysis and evaluation of the classification strategy***

**Fig. 5:**
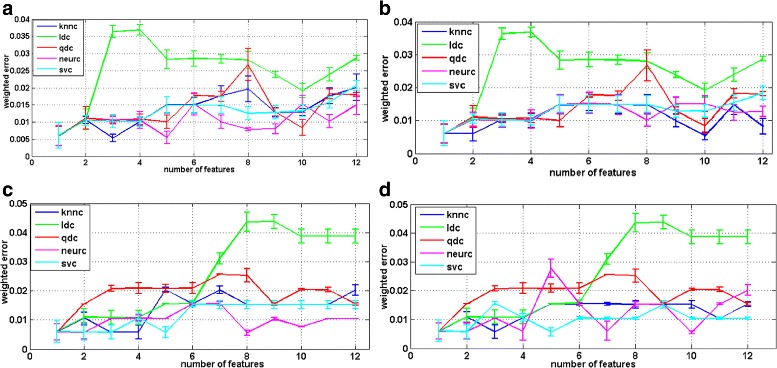
First classifier training (membrane-episode VS super class of cluster-episode and vesicle-episode). **a** Branch & bound feature selection method with standard variance normalization (**b**) Branch & bound feature selection method with within-class variance normalization (**c**) Individual feature selection method with standard variance variance normalization (**d**) Individual feature selection method with within-class variance normalization

**Fig. 6:**
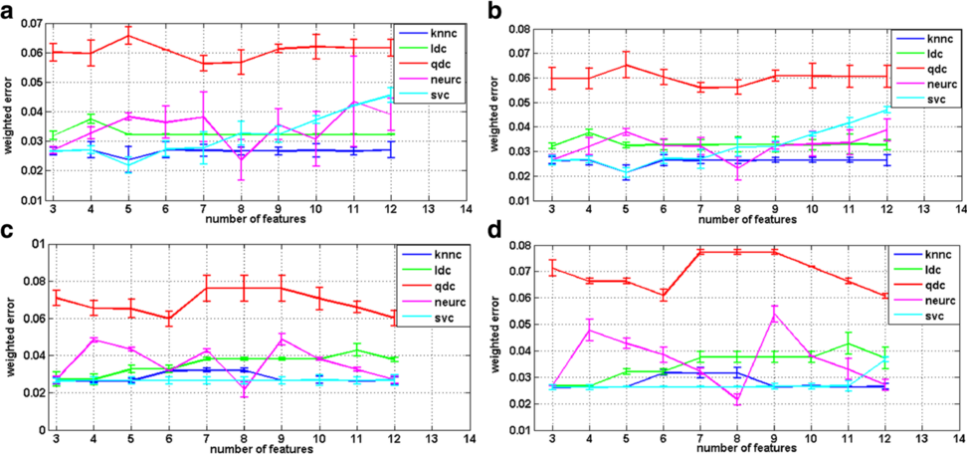
Second classifier training (cluster-episode VS vesicle-episode). **a** Branch & bound feature selection method with standard variance normalization, (**b**) branch & bound feature selection method with within-class variance normalization, (**c**) Individual feature selection method with standard variance variance normalization, (**d**) Individual feature selection method with within-class variance normalization

In order to find a panel of classifiers to address the categorization of our three episode dynamic model, we included two normalization schemes, two feature selection methods and five classifiers. We have presented the results as a weighted error estimation. The classification results are obtained from a weighted error estimation procedure as implemented in PRTools [30]. The results are depicted in Figs. 5 and 6.

The first classification in our scheme should separate the membrane-episode from the super class of the vesicle-episode and the cluster-episode. For this classifier training, i.e. membrane-episode vs. super class of cluster-episode and vesicle-episode, we observe that the weighted error of the linear classifier increases abruptly when the number of features exceeds a certain threshold, i.e. three for branch and bound and eight for individual feature selection method. The weighted error of the K-nearest neighbor classifier is, however, more stable and exhibits the lowest point in the branch and bound feature selection group. The results are summarized in Fig. 5.

**Fig. 7:**
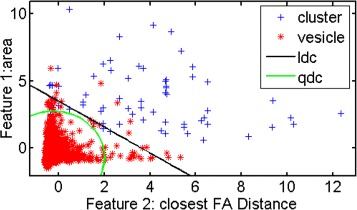
Scatter plot of training data with LDC and QDC. This plot shows the better performance of LDC over QDC for our dataset with two selected features

As for the second classifier training, i.e. cluster-episode vs. vesicle-episode, we noticed, in Fig. 6, the weighted error of quadratic classifier performs worst. We created scatter plots of mapped data with a linear classifier and a quadratic classifier so as to indicate the reasons of the worst performance with the quadratic classifier; depicted in Fig. 7. The error line of the support vector machine classifier is quite stable; it obtains the lowest values in the group using the branch and bound feature selection method. Nevertheless, in the individual feature selection group, the error of neural network classifier always evaluates with the best performance in terms of magnitude of the error.

In order to obtain an overall perspective on the performance, we selected the minimal mean error from all weighted errors with different feature dimensions. This value represents the best performance of the combination between feature selection and classifier. In Table 4, the mean and standard deviation of each minimal value is shown. For the training of the first-step classifier we see that the combination of branch and bound feature selection with the K-nearest neighbor classifier has the lowest minimal value and a relatively small standard deviation with both normalization schemes. For the training of the second-step classifier, the combination of branch and bound feature selection with the support vector machine classifier as well as the combination of individual feature selection with the neural network classifier both have the same lowest minimal value and a comparable small standard deviation in two normalization schemes. Considerations for the combination of the classifiers for the two-step hierarchy that is employed for our application should include generality of the classifier and lower feature dimensions. Therefore we have chosen the combination of branch and bound feature selection with K-nearest neighbor classifier for first step classification using the variance normalization scheme. And, along the same lines, for the second step classification, we have chosen the combination of branch and bound feature selection with the support vector machine classifier using the variance normalization scheme. The motivation for choosing the branch and bound feature selection method is the existence of correlated features on our feature set which causes a much lower performance for the best individual-N feature selection method. The branch and bound feature selection method, on the other hand, guarantees the optimal feature subset without explicitly evaluating all possible feature subsets because the criterion function fulfills the monotonicity condition [37]. The first step of in our two-class classification problem requires a simple but efficient classifier for a basic recognition problem, to that end the choice K-nearest neighbor classifier is efficient. Moreover, a higher value of K provides a smoothness which reduces the vulnerability to noise in the training data. The choice for the support vector machine in the second step is also motivated by its flexibility in the selection of a threshold by introducing the kernel. The support vector machine has, in addition, the ability of maximizing the generalization because it is trained to maximize the margin.

**Table 4:** Weighted error comparison

1st step	knnc		ldc		qdc		neurc		svc	
C-V	mean	std	mean	std	mean	std	mean	std	mean	std
B&B	0.0053	0.0011	0.0061	0.0038	0.0059	0.0028	0.0061	0.0029	0.0061	0.0038
IND	0.0058	0.0023	0.0061	0.0038	0.0059	0.0028	0.0055	0	0.0057	0.0014
2nd step	knnc		ldc		qdc		neurc		svc	
C-V	mean	std	mean	std	mean	std	mean	std	mean	std
B&B	0.0214	0.0031	0.0321	0.0011	0.056	0.0019	0.0231	0.0048	0.0214	0.002
IND	0.0261	0	0.0267	0	0.0607	0	0.0214	0.0021	0.0261	0
1st step	knnc		ldc		qdc		neurc		svc	
VAR	mean	std	mean	std	mean	std	mean	std	mean	std
B&B	0.0053	0.0011	0.0061	0.0038	0.0059	0.0028	0.0055	0.0018	0.0061	0.0038
IND	0.0058	0.0023	0.0061	0.0038	0.0059	0.0028	0.0056	0	0.0058	0.0019
2nd step	knnc		ldc		qdc		neurc		svc	
VAR	mean	std	mean	std	mean	std	mean	std	mean	std
B&B	0.0237	0.0044	0.0319	0.0015	0.0563	0.0027	0.0236	0.0069	0.0218	0.0028
IND	0.0263	0	0.0272	0.0029	0.0598	0.004	0.0218	0.004	0.0265	0.001

C-V represents c-variance

B&B represents branch & bound

IND represents individual

VAR represents variance

**Fig. 8:**
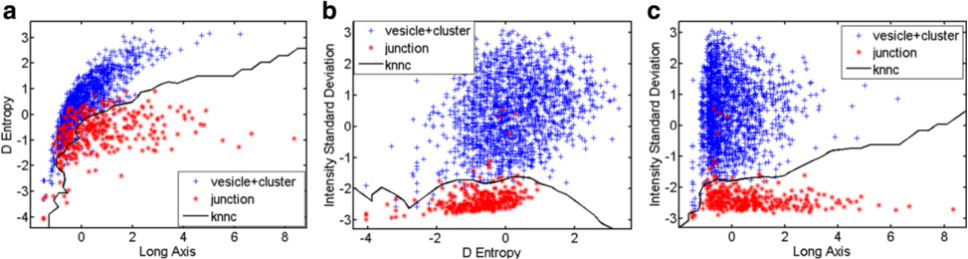
Step 1 scatter plot. For step 1, the KNNC classifier was chosen. In (**a**), (**b**) and (**c**), the performance of the KNNC classifier for the three major features is plotted

For the first step we have chosen the following prominent features: long axis, D-entropy and standard deviation of intensity (cf. Tables 1 and 2). For the second step, we have chosen the top five features; i.e. skewness, entropy, H-entropy, closest object distance and area (cf. Tables 1 and 2). In Table 5 the evaluation of the feature selection performance is shown. The evaluation is done by calculating the probability of each feature being selected by the feature selection method. These selected features best reflect the phenotype changes between three characteristic episodes. For example, in step 1, Long Axis is chosen most of the time because the (plasma) membrane is around the membrane which, in that part of the process, tends to have an elongated shape compared with other two episodes. In step 2, the intensity entropy is selected because clusters have a flatter region compared with the vesicles and this gives rise to a lower entropy value. For both steps of the hierarchical classification scatter plots are depicted in Figs. 8 (step 1) and 9 (step 2). The results of the evaluation articulate that it is not necessary to use large amounts of features. Just a few will contribute to the successful classification results. Moreover, the importance of the wavelet texture features, as stated in the introduction, can be appreciated by their clear contribution to a better performance compared to previous classifier schemes.

**Fig. 9:**
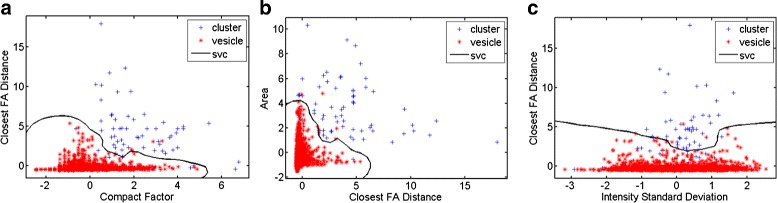
Step 2 Scatter Plot. For step 2, the SVC classifier was chosen. In (**a**), (**b**) and (**c**), the performance of the SVC classifier for the three selected features is plotted

**Table 5:** Feature selection performance

Features	Step 1	Features	Step 2
Long axis	100	Closest FA dist	100
Int Std	100	Int Entropy	100
D_entropy	87	Area	96
H_entropy	7	Int Std	73
V_entropy	5	Compact factor	44
Smoothness	1	Int uniformity	34
Area	0	Smoothness	14
Perimeter	0	H_entropy	8
Extension	0	Border dist/nucleus dist	7
Dispersion	0	Perimeter	6
Elongation	0	Long Axis	6
Orientation	0	Short Axis	5
Compact factor	0	D_std	5
Border dist/nucleus dist	0	Skewness	2
Closest FA dist	0	Extension	0
Short axis	0	Dispersion	0
Skewness	0	Elongation	0
Int uniformity	0	Orientation	0
Int entropy	0	H_mean	0
H_mean	0	H_std	0
H_std	0	V_mean	0
V_mean	0	V_std	0
V_std	0	V_entropy	0
D_mean	0	D_mean	0
D_std	0	D_entropy	0

In order to illustrate and verify the better performance of the hierarchical classification strategy, we conducted an experiment using a single-step multi-class classification strategy on the same dataset. Furthermore, at the same time the necessity of the feature selection procedure was assessed by comparing the performance of a full set of features to a set of selected features obtained by a feature selection procedure. The effect of the feature selection is evaluated for both the single-step multi-class classification strategy and hierarchical classification strategy. We compared the different classifiers that we have used for the hierarchical strategy; i.e. LDC, QDC, KNNC and SVC. For each of these classifiers the results after 50 repetitions for the best performing classifier for each experimental group are listed in Table 6. For the asssessment of the accuracy of an hierarchical classification another measure metric is used. To that end, we have adopted the metric suggested in [6], i.e. the hierarchical f-measure (hF) which is the hierarchical adaptation of the F1 metric in flat classification. The hF is derived from the hierarchical precision (hP) and the hierarchical recall (hR) which are respectively defined as follows:

(3) $$ hP=\frac{\sum_{i} \mid \hat{P_{i}} \cap \hat{T_{i}} \mid }{\sum_{i} \mid \hat{P_{i}} \mid } \qquad\qquad hR=\frac{\sum_{i} \mid \hat{P_{i}} \cap \hat{T_{i}} \mid }{\sum_{i} \mid \hat{T_{i}} \mid }  $$

**Table 6:** Feature selection performance

	F-25	F-sel	H-25	H-sel
hF	0.9603	0.9745	0.9839	0.9889
*σ*	0.0094	0.0103	0.0066	0.0065

hF: F-measure for hierarchical classification

F-25: flat classification with total 25 features

F-sel: flat classification with selected features from branch & bound feature selection method

H-25: hierarchical classification with total 25 features

H-sel: hierarchical classification with selected features

$\hat {P_{i} }$ is the set of predicted class(es) with all of its ancestors and $\hat {T_{i} }$ is the set of real class(es) with all of its ancestors. Subsequently, the f-measure for hierarchical classification is defined as:

(4) $$ hF=\frac{2 \ast hP \ast hR}{hP + hR}  $$

The performance evaluation listed in Table 6 represents the mean of the summations of 215 randomly selected test data set with 100 times repetition for each classification strategy. The F-measure (hF) is given for hierarchical classification as well as flat classification in order to appreciate the improvement of the accuracy. In addition, in Table 6, for all classifications the f-scores (F1) are given and these show the same trend as hF. Furthermore, a test for statistical significance is performed using the two-sample Kolmogorov-Smirnov test [38]. This shows that all f-measures from different classification strategies are significantly different from each other at the 5 % level of significance as shown in Table 7. In addition, in Table 8, the confusion matrices for each approach are presented. For each classification strategy, the confusion matrix is listed in the context of the three class outcome.

**Table 7:** Kolmogorov-Smirnov test result

	F-25 vs F-sel	F-25 vs H-25	F-25 vs H-sel	F-sel vs H-25	F-sel vs H-sel	H-25 vs H-sel
*H*_value	1	1	1	1	1	1
*P*_value	3.3e-17	1.3e-38	3.9e-41	4.4e-15	6.7e-22	5.2e-08

On the basis of hF, all test results render to be significantly different i.e. *H* value is 1

**Table 8:** Confusion matrices for all strategies

[1]		[2]			[3]	[4]
F-25		Prediction			Number of test objects	Sensitivity
		Membrane	Vesicle	Cluster		
Truth	Membrane	34.96	2.04	0	37	0.945
	Vesicle	1.81	166.01	0.18	168	0.988
	Cluster	0.12	4.39	5.49	10	0.549
F-sel		Prediction			Number of test objects	Sensitivity
		Membrane	Vesicle	Cluster		
Truth	Membrane	35.67	1.33	0	37	0.964
	Vesicle	0.58	166.79	0.63	168	0.993
	Cluster	0.17	2.77	7.06	10	0.706
H-25		Prediction			Number of test objects	Sensitivity
		Membrane	Vesicle	Cluster		
Truth	Membrane	34	3	0	37	0.919
	Vesicle	0.11	166.83	1.06	168	0.993
	Cluster	0.07	4.51	5.42	10	0.542
H-sel		Prediction			Number of test objects	Sensitivity
		Membrane	Vesicle	Cluster		
Truth	Membrane	35.53	1.47	0	37	0.960
	Vesicle	0.75	166.86	0.39	168	0.993
	Cluster	0	2.58	7.42	10	0.742

Column 1 represents the strategy and class labels

Column 2 represents the prediction

In column 3, the number of test objects represents the ground truth

In column 4, the sensitivity is given per class

### EGFR endocytosis regulator identification

**Fig. 10:**
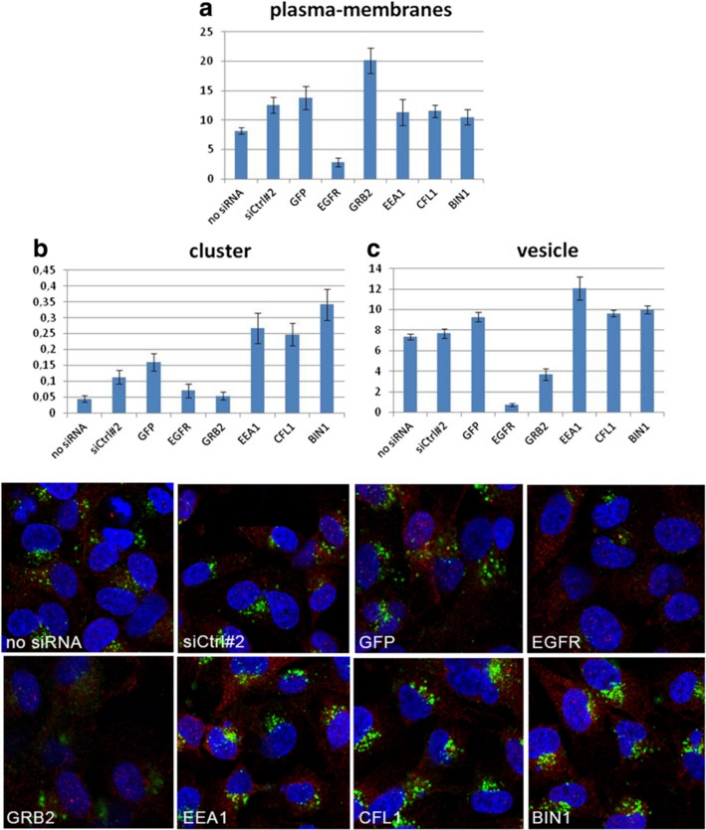
EGFR endocytosis regulator identification results. **a** Pixels of plasma-membrane, (**b**) Number of clusters, (**c**) Number of vesicles

Our hierarchical classification strategy was used to validate siRNA-mediated knock-down of several known EGFR endocytosis regulators; i.e. siGrb2, siEEA1, siCFL. In order to study the effect of the gene knock down, 10 sample points per well were selected for: WT cells (not treated with siRNA), control siRNA treated cells (siCtrl#2 and siGFP), siEGFR treated cells and three target siRNAs. After image processing and data analysis, we calculated the number of objects belonging to each episode group per nucleus and compared the result. These numbers are depicted in Fig. 10. As expected, cells treated with siEGFR show a decreased level in all three episodes since treatment of cells with siEGFR results in > 90 % knock-down of EGFR. Cells incubated with siGrb2 show a drastic reduction in number of endosomes (vesicle-episode and cluster-episode) because siGrb2, as a known regulator of EGFR endocytosis, can significantly inhibit EGFR internalization [39]. In general the increase in number of endosomes (vesicle-episode and cluster-episode) can be caused either by an enhanced uptake of EGFR resulting in an enhanced EGFR endocytosis and EGFR degradation, or by delayed endocytosis and EGFR breakdown. For EEA1, a member of the early endosomes, an increase in number of endosomes (vesicle-episode and cluster-episode) is due to delayed endocytosis [40]. Cofilin (CFL) regulates the cytoskeleton and because of these subsequent changes in the actin cytoskeleton the endocytosis route of EGFR changes [41]. At present, only little knowledge about BIN1 in EGFR endocytosis is available. However, regarding the result, it is observed that BIN1 depletion decreases EGFR at plasma-membrane, increases the number of vesicles and clusters, suggesting that it potentiates EGFR endocytosis and possibly signaling. These results demonstrate the robustness of our hierarchical classification scheme and the capability to predict new EGFR endocytosis regulators.

### Dynamic EGFR endocytosis stage

**Fig. 11:**
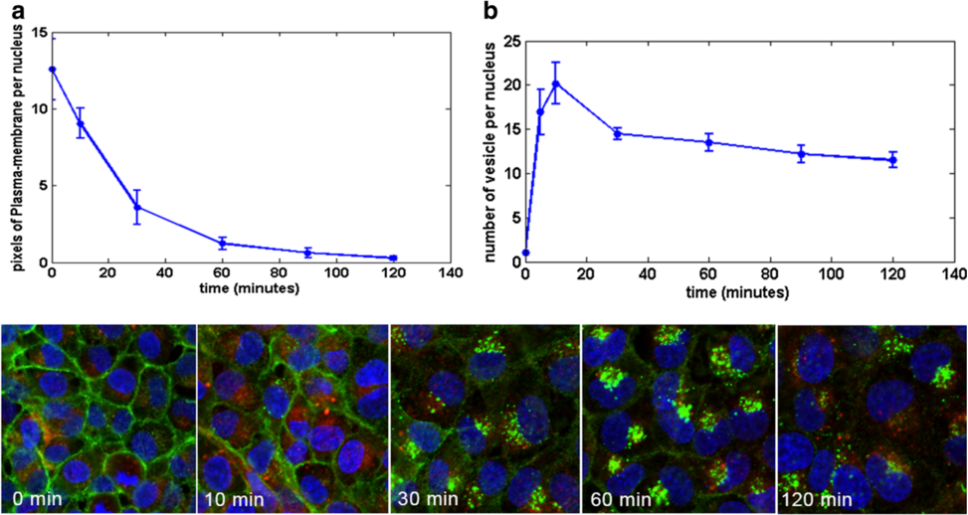
Dynamic stages of EGFR endocytosis. **a** Number of EGFR localized at Plasma-membrane (pixels/nucleus), (**b**) Number of EGFR as vesicle in early endosome (number/nucleus)

In this case study, HBL100 cells were exposed to EGF (50 ng/ml) for indicated timepoints (cf. Fig. 11c). The number of EGFR localizations at the plasma-membrane and the vesicles was quantified. The amount of EGFR localized at the plasma-membrane, expressed as pixels of plasma-membrane per nucleus, decreases over time as shown in Fig. 11a. This fits with the EGFR endocytosis process during which EGF exposure is causing a gradual EGFR re-distribution from the plasma-membrane into vesicles. Meanwhile, the number of vesicles per nucleus increases at the early endosome stage caused by the EGFR internalization, then decreases at the late endosome stage when the vesicles form into a larger complex clusters and degrades at the end as illustrated in Fig. 11b. These graphs indicate the trend of EGFR endocytosis process and are representative in illustrating the dynamics of EGFR endocytosis stages.

## Conclusions

This paper discusses an integrated image and data analysis system for high-throughput screening including wavelet-based texture measurements and a hierarchical classification strategy. From previous studies we have learned [14] how the phenotype description could be improved with new texture features as well as an improved classification of the characteristic episodes with an alternative classification scheme.

For the image analysis we use an innovative image segmentation algorithm combined with representative phenotype measurements which include relative texture features from the wavelet transform to replace the absolute intensity feature so as to decrease the impact of variations in fluorescent intensity between the samples. For the data analysis, we change from a single-step multi-class classification solution into a two step Hierarchical classification strategy to categorize three dynamic phenotypes of the EGFR endocytosis process. We include two feature normalization methods, two feature selection methods and five classifiers to find the best classification strategy. After evaluation of different combinations, we have chosen the combination of branch and bound feature selection with K-nearest neighbor classifier for first step classification after a normalization of variance. As shown in Table 4, the combination of branch and bound feature selection with support vector machine classifier is chosen for the second step classification after having applied the same normalization method.

With the selected combination, the classifier shows a notable improvement in distinguishing the membrane-episode in the data set. This improvement is due to three factors. First, it benefits from the hierarchical classification scheme which introduce multilevel classifiers to deal with two subset classes at a time. Second, we introduced the exact prior probability for the classifier training which improves the performance of the classification strategy significantly. Third, the relative texture measurements show their potential to describe the phenotype characteristics.

This explicit hierarchical classification solution can identify the characteristic episodes in the EGFR endocytosis process and it has shown to be able to support the identification of new regulators in this crucial process relating to breast cancer progression. With all kinds of phenotype measurement and flexible classifier training strategy that we introduced in this study, it has become easier to detect morphological changes of the phenotype and therefore to extend our solution to cope with studies utilizing fluorescence microscopy in a siRNA based high-throughput screening (HTS).
